# Challenging Unsustainable Practices: A Cross‐Sectional Study of Nurses’ and Midwives’ Attitudes Toward Sustainability and Climate

**DOI:** 10.1155/jonm/8947407

**Published:** 2026-08-31

**Authors:** Angela Flynn, Clare Crowley, Mohamad M. Saab, Geraldine Lee

**Affiliations:** ^1^ School of Nursing and Midwifery, University College Cork, Cork, Ireland, ucc.ie

## Abstract

**Introduction:**

Climate change and environmental degradation are increasingly recognised as major global health threats. This necessitates adaptation within health systems and urgent development of mitigatory measures. Nurses and midwives are central to the environmental impact of healthcare and to advancing sustainable practices. This study explored nurses’ and midwives’ attitudes toward sustainability and climate change and perceived barriers to sustainable practice.

**Method:**

A cross‐sectional survey design was used, employing the Sustainability Attitudes in Nursing Survey (SANS‐2) instrument to assess attitudes to sustainability and climate change. The survey included nine core Likert‐scale items (1 = *strongly disagree* to 7 = *strongly agree*) and five further researcher‐designed questions. A convenience and snowball sample of nurses, midwives and students was recruited via professional networks and social media across Ireland, the United Kingdom, Germany, and other European countries. Quantitative data were analysed using descriptive statistics and analysis of variance (ANOVA) to explore differences by professional role. Open‐ended responses were examined using content analysis to identify recurring themes.

**Results:**

There were 256 complete responses. Most participants agreed that climate change (78%) and sustainability (87.1%) are important issues for nursing and midwifery. The majority supported their inclusion in nursing and midwifery curricula (climate change: 68.8%; sustainability: 77.7%). While most participants reported applying sustainability principles at home (84.4%), only just over half reported doing so in their nursing and midwifery practice (53.5%). Significant relationships were found between sustainable behaviours at home and work (*p* < 0.001) and between perceived time availability and belief in climate change importance (*p* ≈ 0.001).

**Discussion:**

Findings reveal strong recognition of sustainability’s relevance but limited translation into practice, particularly among students and early‐career staff. Hierarchical structures and a lack of confidence inhibited challenges to unsustainable practices. Educational reform embedding sustainability within nursing curricula, aligned with the UN Sustainable Development Goals, is essential to empower nurses and midwives as climate health advocates.

**Implications for Nursing Management:**

Nurse managers and leaders should embed sustainability within clinical governance, quality improvement, staff development and leadership practice. Creating psychologically safe environments where nurses can raise concerns about unsustainable practices, alongside visible leadership support and targeted education, may facilitate the translation of positive sustainability attitudes into environmentally responsible practice.

## 1. Introduction

There is increasing evidence of the impact of climate change and the ecological crisis on the health of populations around the world [1]. The impact of poor environmental conditions on health includes challenges such as new and diverse vectors for disease, extreme weather conditions, floods, extreme heat, food and water scarcity and air pollution. These impacts require adaptation of how we practice healthcare as well as rapid development of mitigatory measures. In the case of cardiovascular care, for example, calls have been made to urgently ‘develop and implement guidelines to manage heat‐related presentations’ [2], p101374. Naturally, these impacts are relevant for nursing and for the widening roles that nurses will play in countering and mitigating efforts, in what Schenk and Johnson [3] have referred to as ‘environmental stewardship’ (p.4379).

It is acknowledged that healthcare, as an activity, has a significant carbon footprint [4]. Estimates suggest that healthcare is responsible for 4%–5% of global greenhouse gas emissions [5]. Health services around the world have developed strategies to reduce their carbon emissions with ambitious targets such as achieving net‐zero emissions by 2050 [6–9]. Nurses and midwives make up the largest proportion of health service workforce. They are predominantly on the frontline of healthcare activities and may be seen as contributing to these emissions [10]. With this in mind, it is important to understand the views and attitudes of nurses and midwives to sustainability and to the climate crisis.

## 2. Background

The trusted position of nurses and midwives within healthcare organisational ecosystems places them in a very privileged position in terms of influencing policy. Many of the decisions with the greatest environmental impact such as vehicular fleet modes, source of energy supplies to hospitals, sustainable procurement decisions and waste disposal contracts may seem to be beyond the remit of frontline staff [11]. However, nurses and midwives have an important role to play in influencing decisions that can shape healthcare’s contribution to carbon emissions and can be critically important to more sustainable decision‐making.

Examples of such decisions include nursing involvement in procuring compostable aprons [12], creating a tool for sustainable procurement [13] and choosing reusable versus disposable incontinence products [14, 15]. To ensure that nursing is well positioned to advocate for more sustainable clinical and procurement practices, it is essential to ascertain nurses’ attitudes toward sustainability. Nurse and midwife education has a central role in embedding sustainability concerns and practices into undergraduate education so that future professionals are equipped to advocate for more sustainable decisions and to be effective climate activists in their everyday practices [16, 17]. To address these issues, this research aimed to explore nurses’ and midwives’ attitudes toward climate change and sustainability and the barriers to sustainable practice. These findings will help inform how we prepare nurses and midwives to practice more sustainably.

Despite growing recognition of the healthcare sector’s contribution to climate change and environmental degradation, empirical evidence on nurses’ and midwives’ attitudes toward sustainability remains limited. The Sustainability Attitudes in Nursing Survey (SANS‐2) has been used in several studies to assess sustainability awareness among nursing populations [18–20]. However, most studies have focused on single professional groups, institutions or countries, limiting understanding of how sustainability attitudes vary across the broader nursing and midwifery workforce.

Nurses, midwives, educators, managers and students occupy distinct roles within healthcare systems and influence clinical practice, leadership and professional education Salas et al., 2021; [23]. Yet, sustainability attitudes across these groups have rarely been examined together. Understanding these perspectives collectively may provide a more comprehensive picture of the profession’s readiness to support environmentally sustainable healthcare.

Addressing climate change within healthcare is not solely an individual professional responsibility but also an organisational and leadership challenge. Nursing leaders, educators and managers play a critical role in shaping institutional policies, allocating resources and embedding sustainability within clinical practice, governance and professional education. Understanding sustainability attitudes across different professional roles may therefore provide insights into the profession’s capacity to support organisational change toward environmentally sustainable healthcare systems.

To address the abovementioned gaps, the aim of this study was to examine attitudes toward sustainability and climate change among nurses, midwives, students, educators and managers across multiple European countries using the SANS‐2 instrument and to explore how these attitudes vary across professional roles that influence the implementation of sustainable healthcare practices. This study is conceptually informed by the theory of planned behaviour [25], which proposes that attitudes influence behavioural intentions and subsequent actions, while contextual factors shape individuals’ ability to enact those behaviours. In healthcare settings, the translation of sustainability attitudes into practice may also be influenced by professional role, decision‐making authority and organisational constraints. For example, managers and educators may have greater influence over institutional policies and curricula, whereas clinicians and students may experience more limited control over organisational practices. Understanding attitudes toward sustainability across these professional groups may therefore provide insights into the profession’s capacity to support environmentally sustainable healthcare practices within existing organisational structures.

## 3. Materials and Methods

### 3.1. Study Design

Building on the work of Álvarez‐Nieto et al. [26] and Richardson et al. [20], this cross‐sectional study collated the views of nursing and midwifery professionals and students in relation to sustainability practices both at home and at work using the SANS‐2 instrument [26].

### 3.2. Sample and Procedures

Convenience and snowball sampling strategies were used to recruit nurses, midwives and nursing or midwifery students. Recruitment was conducted via practitioners and students within the nursing and midwifery professions. Networks of nursing and midwifery organisations, including those with an interest in sustainability, were asked to share the link to the online survey. Social media platforms were also used to share the survey link to across nursing and midwifery social networks. While these recruitment strategies enabled the inclusion of participants from multiple countries and professional roles, they may have increased the likelihood of participation among individuals with a preexisting interest in sustainability or climate‐related issues.

The online survey was hosted in Qualtrics and was open for responses from March to August 2023. The SANS‐2 is widely used to investigate attitudes toward sustainability in nursing cohorts [27]. It has also been used to assess the impact of educational interventions relating to sustainability on nursing student cohorts in multiple countries including the United Kingdom, Germany, Spain and Switzerland [20, 28]. The SANS‐2 comprises nine items using a 7‐point Likert scale ranging from ‘*strongly disagree*’ to ‘*strongly agree*’. Five further researcher‐designed questions were included in the current study. Of those, one question explored the concept of challenging unsustainable practices, informed by Aronsson et al. [29], and the remaining four captured barriers to healthcare being more sustainable, time for engagement with sustainability practices, participants’ role and years of practice. Internal consistency reliability was assessed in the present study using Cronbach’s alpha. The SANS‐2 demonstrated good internal consistency (*α* = 0.78). The data collection instrument can be found in Table 1.

**TABLE 1:** Data collection instrument.

Question	Source
Q1‐Climate change is an important issue for nursing and midwifery	SANS‐2
Q2‐Issues about climate change should be included in the nursing and midwifery curricula	SANS‐2
Q3‐Sustainability is an important issue for nursing and midwifery	SANS‐2
Q4‐Sustainability should be included in the nursing and midwifery curricula	SANS‐2
Q5‐I apply sustainability principles at home	SANS‐2
Q6‐I apply sustainability principles in my nursing/midwifery practice	SANS‐2
Q7‐I am aware of unsustainable practice in my work environment	SANS‐2
Q8‐I challenge unsustainable practice in my work environment	SANS‐2
Q9‐I feel unable to challenge unsustainable practice in my work environment	SANS‐2
Q10‐If you feel unable to challenge unsustainable practice, could you briefly explain why you have felt unable to challenge practice?	Researcher‐designed, informed by Aronsson et al. [29]
Q11‐What is your job/role?	Researcher‐designed
Q12‐How many years have you been in practice?	Researcher‐designed
Q13‐What barriers do you think prevent healthcare being more sustainable?	Researcher‐designed
Q14‐Do you feel you have time to engage in sustainability practices?	Researcher‐designed

### 3.3. Ethics Statement

Ethical approval for the study was granted by the Social Research Ethics Committee of University College Cork (Log 2023‐059). Informed consent was gained from all participants who received detailed information regarding the study’s purpose. It was made clear that participation was voluntary. All data were collected anonymously, and no identifying information was captured.

### 3.4. Data Analysis

Quantitative data collected through the SANS‐2 were analysed using descriptive and inferential statistical methods. All survey responses were exported from Qualtrics to *R* and cleaned to remove incomplete entries. Survey responses were considered incomplete if more than 14% of items were missing (*n* = 97 cases excluded entirely). However, partial responses were retained for analysis when they contained complete data for the specific analyses of interest (e.g., early SANS‐2 items Q1–Q9 for attitude summaries or Q10–Q14 for barrier themes). This approach maximises the use of available data from a hard‐to‐reach convenience sample while minimising bias from differential attrition. To assess potential impact, we conducted a sensitivity analysis comparing key descriptives (median SANS‐2 scores and role distributions) between complete‐case (*n* = 256) and all‐available‐case analyses; results were substantively identical (largest absolute difference in medians: 0.2 scale points), supporting the robustness of retaining partials.

SANS‐2 items are ordinal Likert‐type responses (Q1–Q9). We report medians and interquartile ranges (IQRs) as primary descriptors of central tendency and variability, with frequencies/percentages for categorical variables (role and years of experience). Associations between categorical variables were examined using chi‐square tests (*p* ≤ 0.05; Cramér’s V for effect size). Although technically ordinal, mean scores across Q1–Q5 were also calculated and compared across professional groups (nurse/midwife, academic, student and manager) using one‐way ANOVA, following common practice for approximately symmetric multi‐item scales. Normality, homogeneity of variance and post hoc tests were evaluated as appropriate. These parametric analyses complement the ordinal summaries and are interpreted cautiously. Cross‐study comparisons were made using published mean scores from prior research [26, 30, 31] to contextualise findings from student participants.

Open‐ended responses to Q10, 13 and 14 were examined using qualitative content analysis [32]. Responses were exported from Qualtrics, read repeatedly by the first and second authors and initially coded using descriptive labels that captured the manifest content of each statement (for example, ‘time constraints’, ‘hierarchy’, ‘lack of organisational support’ and ‘resource limitations’). Codes with similar meaning were then grouped into broader categories (for example, ‘time and workload pressures’, ‘limited authority to influence change’ and ‘structural and organisational barriers’), which we report as overarching themes in the Results. This approach allowed us to summarise recurring barriers and perceived obstacles to sustainable practice in a large international survey dataset.

## 4. Results

### 4.1. Sample Characteristics

The online survey was accessed on 353 occasions. Of those, 97 responses were incomplete. While some participants did not complete all sections of the survey, many provided valid and complete responses to the earlier questions. These partial responses were retained for analysis where the relevant data were available. Complete‐case and available‐case analyses were used as appropriate. Retaining partial responses helped preserve sample size and reduce bias, as noncompletion was not systematically related to earlier answers. Full survey responses were completed by 256 participants from 13 countries (Australia, Belgium, Canada, Germany, Ireland, Italy, Portugal, Kenya, Switzerland, Spain, South Africa, Turkey and the United Kingdom). Participants were nurses (*n* = 81, 31.6%), nursing/midwifery academics (*n* = 60, 23.4%), nursing/midwifery students (*n* = 45, 17.6%), nursing/midwifery managers (*n* = 22, 8.6%) and midwives (*n* = 6, 2.3%). A total of 10 (3.9%) participants selected ‘other’ for their role, and 32 (12.5%) did not specify their role. In total, 220 participants reported their years of experience, which ranged widely from 0 to 48 years, with a median (IQR) of 19 years (5–30).

### 4.2. Attitudes Toward Sustainability and Climate Change

The majority (*n* = 200, 78.1%) of participants agreed that climate change is an important issue for nursing and midwifery with 176 (68.8%) agreeing that climate change should be included in the nursing and midwifery curriculum. A greater proportion (*n* = 223, 87.1%) of participants agreed that sustainability is an important issue for nursing and midwifery, and the majority (*n* = 199, 77.7%) agreed that sustainability should be included in the nursing and midwifery curriculum. Most participants (*n* = 216, 84.4%) agreed that they apply sustainability principles at home, with fewer (*n* = 137, 53.5%) agreeing that they apply sustainability principles in their nursing and midwifery practices. The median (IQR) scores for all Likert‐scale items of SANS‐2 are presented in Table 2.

**TABLE 2:** Median scores for Likert SANS‐2 questions.

Question	*n* ^∗^	Median (IQR)
Climate change is an important issue for nursing and midwifery	264	6 (5–7)
Issues about climate change should be included in the nursing and midwifery curricula	264	6 (4–7)
Sustainability is an important issue for nursing and midwifery	262	6 (5–7)
Sustainability should be included in the nursing and midwifery curricula	262	6 (5–7)
I apply sustainability principles at home	255	6 (5–7)
I apply sustainability principles in my nursing/midwifery practice	254	5 (4–6)
I am aware of unsustainable practice in my work environment	240	6 (5–7)
I challenge unsustainable practice in my work environment	240	4 (3–5)
I feel unable to challenge unsustainable practice in my work environment	240	4 (2–6)

^∗^Available‐case analysis was used as appropriate.

When exploring the relationship between the different variables, we found no statistically significant relationship between awareness of unsustainable practices in the work environment and the number of years in practice. A strong statistically significant relationship was observed between those who reported applying sustainability principles in their nursing/midwifery practice and those who applied them at home (*p* < 0.001), suggesting consistency in sustainable behaviours.

There was a statistically significant relationship between those who felt they had time to engage in sustainability practices and those who believed climate change is an important issue for nursing and midwifery (*p* ≈ 0.001, Cramér’s V = 0.347), indicating that those with stronger climate concern are more likely to make time for sustainable actions (Table 3). Additionally, a statistically significant relationship was found between those who reported applying sustainability principles at home and those who were aware of unsustainable practices in their work environments (*p* = 0.003), suggesting that personal sustainability practices may enhance professional awareness.

**TABLE 3:** The relationship between Q1 and Q14.

		Q1: Climate change is an important issue for nursing and midwifery							
		Total	1 *strongly disagree*	2 *disagree*	3 *somewhat disagree*	4 *neither agree nor disagree*	5 *somewhat agree*	6 *agree*	7 *strongly agree*
Q14: Do you feel you have time to engage in sustainability practices?	Total count (answering)	185	6	5	14	18	27	22	93
	Yes	116	2	1	7	10	10	16	70
		62.7%	33.3%	20%	50%	55.6%	37%	72.7%	75.3%
	No	69	4	4	7	8	17	6	23
		37.3%	66.7%	80%	50%	44.4%	63%	27.3%	24.7%

Participants who reported applying sustainability principles in their professional practice were also more likely to believe that sustainability should be included in nursing and midwifery curricula. This was supported by a statistically significant relationship between responses to ‘I apply sustainability principles in my nursing/midwifery practice’ and ‘Sustainability should be included in the nursing and midwifery curricula’ (*p* < 0.001).

While exploring the mean scores by role, the lowest SANS‐2 mean score was among students (mean 4.91 [SD 1.24]) and highest among midwives (mean 6.17 [SD 0.82]) (Table 4).

**TABLE 4:** SANS‐2 (Q1–Q5) mean scores by role.

Roles	*n*	Mean (sd)
Overall	256	5.69 (1.22)
Nurse	81	5.84 (1.13)
Academic	60	5.80 (1.28)
Student	45	4.91 (1.24)
Manager	22	5.83 (1.14)
Midwife	6	6.17 (0.82)
Other	10	5.64 (1.34)
Not stated	32	6.06 (1.04)

### 4.3. Awareness of and Challenging Unsustainable Practices

Of 240 participants who responded to this question, a large proportion agreed that they were aware of unsustainable practices in their work environment (*n* = 181, 75.4%). A similar, but reversed, question asked participants if they felt unable to challenge unsustainable practice in their work environment. This question yielded similar results that confirm the earlier finding of a reluctance to challenge unsustainable practices.

More junior staff were less likely to report challenging unsustainable practices. Among the 19 participants who strongly disagreed with the statement *‘I challenge unsustainable practices in my work environment,’* 12 (63.2%) had been in practice for less than five years, suggesting that newer professionals may feel less confident or empowered to take action. This pattern was echoed in responses to the statement *‘I feel unable to challenge unsustainable practices’* where 53% of those who strongly agreed (*n* = 37) also had limited experience. Additionally, 28% of participants with ten years or less of practice strongly disagreed with the statement.

An open‐ended text question asked participants to expand on why they felt unable to challenge unsustainable practices. A small number of participants (*n* = 10, 9.6%) referred explicitly to *time* acting as a constraint to more sustainable practices with statements such as those shown in Table 5. The issue of time is explored further in the responses to Q14.

**TABLE 5:** Illustrative categories from content analysis: impact of time on challenging unsustainable practices (*n* = 104 responses).

If you feel unable to challenge unsustainable practice, briefly explain why you have felt unable to challenge practice
‘Falls on deaf ears, esp and surprisingly with the younger generation. They ‘tolerate’ my pointing out ways we can play our part but don’t tend to follow suit. Also don’t see it as high up on management’s priority list unless it means saving money. […] haven’t the time to pursue it in earnest’
‘Time pressures lack of control over supplies, heating etc’
‘There are very few people trying to raise awareness, and this event requires the intense effort and serious time of a large number of individuals’
‘Lack of resources and time. Not considered a priority in the clinical setting’
‘Busy and worn out at times from repeating the same things’
‘It costs more time. efficiency rules in the hospital’

A number of participants indicated that a perceived lack of seniority or authority prevented them from challenging unsustainable practices (Table 6).

**TABLE 6:** Illustrative categories from content analysis: impact of authority/power on challenging unsustainable practices (*n* = 104 responses).

If you feel unable to challenge unsustainable practice, briefly explain why you have felt unable to challenge practice
‘I have not enough authority to force change and implement innovations or change in actions’
‘As a student nurse one is only in a ward for a few weeks so therefore one feels as though they don’t have the authority to be questioning things as we are only learning and have very little knowledge as first year students’
‘I can’t go against what my seniors prescribe as it will be taken as insubordination. I have tried to conscientise them about the need of changing our doings to be climate change conscious but to no avail’
‘Feel unsure if it is my place to do so’
‘I don’t feel I have the power to influence this’
‘As a Student I didn’t feel confident enough to ask older colleagues to change their behaviour’
‘Lack of confidence, feel that I will be ignored or made to feel foolish for asking’

### 4.4. Barriers to More Sustainable Healthcare

A key intention of this research was to identify barriers to healthcare being more sustainable. Responses to this question illustrated that time, knowledge and resources were the most cited factors that were perceived as acting as barriers to more sustainable practices in healthcare. A total of 35 participants explicitly referred to knowledge with statements such as ‘*knowledge, attitudes and access to resources*’ and ‘*lack of knowledge on issues of sustainability*’. A number of participants linked the lack of knowledge to lack of education and time ‘*High workload paired with time constraints. Limited education and knowledge around sustainability in healthcare*’. Other participants referred to wider need for institutional support, for example, *‘Lack of education, interest, effort, managerial support and real policies around sustainability in healthcare’*, and ‘*Habits, lack of knowledge, lack of understanding of the magnitude of the consequences, lack of global vision, competing priorities, management with different agenda’.*

A number of participants (*n* = 19) referred explicitly to single‐use plastics, with statements such as *‘Lack of recyclable packaging. Waste of instruments that could be recycled but are single patient use’* and *‘Affordability of solutions. Provenance of single use products contribute to a single use attitude in healthcare environments’.*

The need to balance sustainability with infection prevention measures was also evident from the responses. Responses alluding to this balance included the following: ‘*belief that single use is better for patient and infection control*’; ‘*Single use equipment and infection control measures*’ and ‘*Infection prevention policies = significant barrier. My health board has such complex and extremely hierarchal management structure that slows the progress of everything*’.

The connection between sustainability and infection control aligns with a finding of Chen and Price [30] who found in their study, using the SANS‐2, that nurses’ conceptual understanding of sustainability was focused mostly on environmental aspects and clinical waste management.

### 4.5. Time to Practice Sustainably

Potential for improvement in practice is evident from the results of the final question (Q14) which asked, ‘Do you feel you have time to engage in sustainability practices?’. A strong majority, 63% of participants (*n* = 184), answered yes to this question, suggesting that restrictions on time are not a major factor in preventing more sustainable practices. However, responses to the open questions suggest that time remains a relevant qualitative barrier for a meaningful minority of respondents, as seen in Table 5.

On another positive note, there was a statistically significant relationship between Q14: Do you feel you have time to engage in sustainability practices? and Q1: Climate change is an important issue for nursing and midwifery, indicating that those who most strongly believe climate change is important for nursing and midwifery were of the belief that they did have time to engage in sustainability practices. This finding suggests that those with strong feelings regarding climate change are positively minded and may seek to carve out what they see as essential time to practice more sustainably.

## 5. Discussion

This study aimed to examine the attitudes of nurses and midwives to sustainability and climate change and identify barriers to more sustainable healthcare practices. The findings can inform the curricula for nurses and midwives. The results indicate strong beliefs about the importance of sustainability and climate change in relation to healthcare and therefore inclusion in curricula. Our findings are consistent with earlier SANS‐2 studies in suggesting generally positive attitudes toward climate change and sustainability among nursing cohorts, alongside gaps between attitudes and reported practice [20, 26, 31]. Prior work in single‐country samples has also reported high endorsement of the importance of sustainability and climate curricula, with variability in translation to clinical behaviours [27, 31]. Given substantial differences in sampling frames and national contexts, we do not attempt formal cross‐study comparisons but instead use these studies to situate our findings within the emerging literature.

Given the large proportion of participants who were aware of unsustainable practices at work, it would be important that nurses and midwives feel they were able to take actions to challenge these unsustainable practices. However, the results indicate some uncertainty among participants in relation to challenging unsustainable practices in their work environment.

A reluctance to challenge unsustainable practices was a surprising finding from this study. The responses indicate the preponderance of hierarchical structures in healthcare settings which may have been thought to be outdated. These findings suggest that there is still reluctance among those who witness unsustainable practices to challenge these with some fearing repercussions from more senior colleagues. This concern seems to be expressed most frequently among students and novice newly qualified staff, reinforcing the idea that novice nurses and midwives may face barriers—such as lack of authority or confidence—when it comes to challenging unsustainable practices in clinical settings.

The recurring emphasis on hierarchy, lack of authority and fear of challenging senior staff aligns with Edmondson’s [33] framework of psychological safety—the shared belief that the team is safe for interpersonal risk‐taking. In low‐safety environments, even staff aware of unsustainable practices (71% in this study) hesitate to speak up, particularly junior nurses and students, who cited lack of seniority as a barrier. This suggests that healthcare’s traditional power gradients suppress sustainability advocacy, mirroring patterns observed in patient safety ‘speaking up’ failures [34]. Our multinational sample reinforces that these dynamics transcend national contexts, highlighting a systemic cultural challenge.

Our findings suggest that while knowledge and awareness of sustainability are important, they are not sufficient to enable nurses and midwives to challenge unsustainable practices. Given that participants most frequently described barriers related to hierarchical workplace cultures, fear of repercussions and perceived lack of authority, this indicates that the problem is primarily structural rather than simply educational. Educational interventions therefore need to be coupled with organisational and policy changes that redistribute authority, legitimise speaking up and provide visible leadership support for sustainable practice.

In keeping with the theory of planned behaviour [25], participants demonstrated highly positive attitudes toward sustainability, with most recognising its importance for nursing and midwifery and supporting its inclusion in professional education. However, these positive attitudes did not consistently translate into sustainable practice, suggesting the influence of the remaining theory constructs. The qualitative findings indicate that subjective norms, particularly hierarchical workplace cultures and the perceived expectations of senior colleagues and managers, influenced participants’ willingness to challenge unsustainable practices. Similarly, perceived behavioural control emerged as a significant barrier, with participants describing limited authority, lack of confidence, insufficient organisational support and competing workload demands as obstacles to acting sustainably.

To support organisational change, education remains a necessary, but not a standalone, strategy. Undergraduate and continuing professional education can help nurses and midwives to develop the language, confidence and ethical framing to question unsustainable practices. However, without parallel efforts to address hierarchical dynamics, clarify formal roles in sustainability and protect staff who challenge entrenched routines, additional education is unlikely to translate into consistent action. Our data on fear, lack of authority and the influence of senior staff highlight the need for interventions that target team culture and leadership alongside curriculum reform.

The specific finding of a statistically significant relationship between those who felt they had time to engage in sustainability practices and those who believed that climate change is an important issue for nursing and midwifery is worthy of further discussion. This finding suggests that those who are aware and alert to the importance of climate change and sustainability issues somehow find the time to practice more sustainably. This finding therefore emphasises the need for nurse education to engage in sustainability education among undergraduate and graduate students in nursing and midwifery.

However, the challenge of carrying the moral burden of healthcare’s impact on the environment should not be underestimated. Positioning already overburdened nurses on the frontline of often under‐resourced and poorly staffed healthcare as being instrumental in mitigating for the existential threat of climate change brings an additional moral burden with the allocation of additional environmental responsibility [15]. To ask nurses and midwives to take on this burden as well as to challenge colleagues or policies that are unsustainable would seem to be a significantly burdensome expectation.

### 5.1. Students

Our sample of nursing and midwifery participants included 45 students (17.6%), enabling comparison with previous studies using the SANS‐2 among student populations (see Table 7). Among these students, the mean SANS‐2 total score was 4.91 (SD 1.24), lower than the 5.37 (SD 1.05) reported by Alvarez‐Nieto et al. [28], suggesting that our participants held more negative attitudes toward sustainability and climate change in clinical practice. Chen and Price [30] reported a higher mean score of 5.80 for nursing students from China, compared with 4.48 for students from the United Kingdom. Cruz et al. [31] found results closer to ours, with Arab nursing students scoring a mean of 5.02 (SD 1.43).

**TABLE 7:** Comparison of mean SANS‐2 scores among students in the current study and previous studies.

Studies	Total SANS‐2 score
Cruz et al. [31]	5.02 (SD = 1.43)
Chen and Price [30]	5.80 (SD 0.55) (China); 4.48 (SD 0.37) (UK)
Alvarez‐Nieto et al. [26]	5.372 (SD = 1.05)
Our study (student responses only)	4.91 (SD = 1.24)

In this multinational sample, students scored lower than practicing professionals across most SANS‐2 items and also lower than student cohorts reported in several prior single‐country studies. Three contextual factors may explain these differences. First, curriculum structures vary widely across European nursing programs, with sustainability often limited to isolated lectures rather than integrated as a core competency across preclinical and clinical years. Second, exposure to sustainability content remains inconsistent; many students reported no formal teaching on healthcare’s carbon footprint or practical strategies for sustainable practice, unlike peers in sustainability‐focused institutions. Third, national variation matters—students in countries with weaker healthcare decarbonisation policies or lower public climate awareness may receive less reinforcement of sustainability as a professional priority during training.

These findings highlight students as a key target for intervention. Curriculum redesign should embed sustainability longitudinally, incorporating mandatory modules on healthcare emissions, waste audits during placements and interprofessional climate change simulations. National nursing bodies could also standardise minimum sustainability competencies to reduce cross‐country disparities in baseline attitudes.

### 5.2. Implications for Nursing Management and Practice

The findings have several implications for nursing management and leadership. While individual attitudes toward sustainability are important, the translation of these attitudes into practice is strongly shaped by organisational structures, leadership priorities and resource allocation. Nursing managers and leaders are uniquely positioned to influence institutional sustainability strategies through policy development, workforce education and integration of environmentally sustainable practices into clinical governance frameworks. Differences in sustainability attitudes across professional roles may therefore highlight opportunities for targeted leadership interventions, including integrating sustainability into nursing curricula, continuing professional development and organisational quality improvement initiatives such as the SusQI framework [35]. Strengthening leadership engagement in sustainability may support the transition toward more environmentally responsible healthcare systems.

Senior nurses and managers must therefore model sustainable behaviours and actively foster psychological safety through visible endorsement of staff challenges to wasteful practices. Leadership training could incorporate sustainability scenarios requiring leaders to practice responding supportively to staff concerns (e.g., ‘That’s a valid point – let’s explore compostable alternatives’). Integrating sustainability into formal leadership competencies, alongside mechanisms like sustainability champions or anonymous reporting channels, would legitimise speaking up and shift responsibility from individuals to organisational systems.

In order to enable and equip nurses and midwives to act as climate activists and to challenge unsustainable practices, there is a need to prepare undergraduates to be well informed and appropriately prepared for practice through their curricula. There is strong evidence acknowledging that nurses and midwives are central to countering the high carbon footprint of healthcare and that behaviours and attitudes can be shaped positively by educational interventions [17]; Moustafa Saleh and Elsabahy 2022. Such interventions will equip graduates to engage in vital ‘environmental stewardship’ described by Schenk and Johnson [3].

A useful framework for structuring nursing’s engagement with sustainably practices, as well as wider societal improvements, can be seen in the United Nations’ Sustainable Development Goals (UNSDGs) [37]. The UNSDGs are seen as a globally understood ambition to ensure sustainable practices and growth without compromising the future of the planet. However, the UNSDGs are often seen as detached from nursing realities and practices [38–40]. However, given the ubiquitous nature of the UNSDG ambitions across national and regional organisations and governments, they serve as a useful vehicle to structure education in a manner that embeds nursing efforts toward sustainability within wider global activities.

Nurse education programmes and modules should therefore utilise the UNSDGs and by mapping curricular content against the goals, nurse education can be answerable to the critically important goals contained within the UN’s sustainable development agenda. To drive and incentivise this, core curricular content on sustainability should be included in the undergraduate nursing programmes and should feature strongly across national nursing standards and codes of practice.

### 5.3. Limitations

Full completion of the survey by all participants was challenged in this study, resulting in a limitation of incomplete surveys. The role of social desirability in responses to sustainability questionnaires also needs to be acknowledged as a potential confounding factor [41]. Moreover, participants were recruited using convenience and snowball sampling, including distribution through social media networks. This approach may have resulted in self‐selection bias, whereby individuals with greater interest in sustainability or climate change were more likely to participate. Consequently, the findings may overestimate positive sustainability attitudes and may not fully represent the broader nursing and midwifery workforce. Future studies using more systematic sampling approaches would help to strengthen the generalisability of the findings.

## 6. Conclusions

This study has provided a deeper understanding of the attitudes of nurses and midwives toward climate change and sustainability. There is a greater need for sustainable practices to be made more feasible within the practice setting. The finding of higher perception of relevance for *sustainability* than for *climate change* is of interest and is worthy of further investigation.

Participants in our study compared favourably in terms of their attitudes toward sustainability and climate change, when we compared our results with other studies using the SANS‐2 instrument. There were good levels of awareness of both sustainability and climate change among the nurses and midwives and students surveyed. However, we should be mindful of the findings indicating that, while participants ranked an issue of importance, this did not always result in an application of the related principles in practice.

Finally, we believe this study has provided us with better insights into the barriers nurses and midwives perceive as preventing them from engaging in more sustainable practices. These findings can be used to inform our preparation and education of nurses and midwives to enable and empower them to practice more sustainably.

## Author Contributions

Angela Flynn: conceptualisation, investigation, instrument development, data collection, formal analysis, writing–original draft and reviewing and editing. Clare Crowley, Mohamad M. Saab and Geraldine Lee: formal analysis and writing–review and editing.

## Funding

No funding was received for this research.

## Conflicts of Interest

The authors declare no conflicts of interest.

## Data Availability

The data that support the findings of this study are available from the corresponding author upon reasonable request.
